# Predatory Journals

**DOI:** 10.31744/einstein_journal/2025ED1663

**Published:** 2025-05-22

**Authors:** Jacyr Pasternak

**Affiliations:** 1 Hospital Israelita Albert Einstein São Paulo SP Brazil Hospital Israelita Albert Einstein, São Paulo, SP, Brazil.

In 2024, approximately 89 research papers on predatory journals were published in PubMed. If you are a physician that publishes academic papers—not necessarily one that publishes many or science transforming ones—you may receive several email invitations to publish in journals that you are not familiar with. Some may have titles that are similar to the renowned journals with a good impact factor index that you hold in high esteem. They promise a rapid review of your manuscript without peer-review.^(1)^

You are the prey that they seek. They will flatter you saying that you are a leader in your field, they would be honored by your collaboration, they understand the problems with publishing; the delays in evaluating your work; critical peer-reviewers, and may claim that their streamlined system can help you publish as soon as you send them the manuscript with the publishing fee.^(2)^

## Why is this phenomenon becoming widespread?

With the increase in open access journals, the publishing industry is losing one of their best funding options: the subscription fees that medical libraries and researchers pay. Publishers have now become dependent on publishing fees. You may have heard the phrase *"publish or perish.*" Some global institutions pay their medical researchers based on how many papers they aim to publish every year. Some academic medical institutions also judge and decide the researchers’ tenure based on the number of papers published.

Many enterprising individuals have discovered an easy way of making money. Some do the minimal work required to imitate an actual journal, with an eminent-looking reviewing body to dupe you into submitting a paper while evidently charging a hefty fee. Others do not even attempt this: they merely wish to get their hands on your paper and your money, and you will never hear from them again. They will not respond to your questions, and you would be unable to trace them. As P. T. Barnum said, "There's a sucker born every minute."

Beware of becoming a prey to such fake journals. Verify the data of any journal that invites you to publish with it. Avoid publishing with anyone desperately seeking your collaboration. Do not fall for flattery. Legitimate scientific journals do reach out but only to real leaders in their field. Ask yourself if you are one. lf not, and most of us are not, then it is most likely a predatory journal on the hunt. And you, my friend, are the prey.^(3,4)^
